# Editorial: Computational Modeling for Liver Surgery and Interventions

**DOI:** 10.3389/fphys.2022.859522

**Published:** 2022-02-15

**Authors:** Bruno Christ, Uta Dahmen, Nicole Radde, Tim Ricken

**Affiliations:** ^1^Cell Transplantation/Molecular Hepatology Lab, Department of Visceral, Transplant, Thoracic and Vascular Surgery, University of Leipzig Medical Center, Leipzig, Germany; ^2^Experimental Transplantation Surgery, Department of General, Visceral and Vascular Surgery, Jena University Hospital, Jena, Germany; ^3^Institute for Systems Theory and Automatic Control, Faculty of Engineering Design, Production Engineering and Automotive Engineering, University of Stuttgart, Stuttgart, Germany; ^4^Institute of Mechanics, Structural Analysis and Dynamics, Faculty of Aerospace Engineering and Geodesy, University of Stuttgart, Stuttgart, Germany

**Keywords:** liver surgery, liver perfusion and function, multi-scale modeling, surgical planning and simulation, systems medicine

The liver is a major organ involved in the maintenance of whole body metabolic homeostasis. The underlying metabolic endeavors are assigned to the parenchymal cells of the liver, the hepatocytes, which cluster in so-called liver lobules. At the entrance side of the hepatic lobule, blood, rich in nutrients, reaches the lobule from the intestine via the branches of the portal vein. At the exit side, the blood leaves the lobules via the branches of the central vein. Hepatocytes lined up along this blood axis, called hepatic sinusoid, are functionally different depending on nutrient, hormone and morphogen gradients forming with the blood stream (Jungermann and Kietzmann, 1996; Gebhardt and Matz-Soja, 2014).

The liver is a highly regenerative organ. Removal (resection) of even more than two thirds of the liver due to tumor removal or living liver donation initiates the restoration of the complete liver mass in 6 to 12 months. However, partial liver resection causes significant changes of the vascular supply, which propagate from the macroscopic anatomical scale down to the cellular microscale of the hepatic sinusoids. This has functional consequences facing the versatile range of hepatic tasks. Describing the complex relation between changes in hepatic blood perfusion and forecast of functional consequences is not trivial. Therefore, complex liver surgery still carries a not negligible risk of post-surgery liver failure.

This Research Topic aims to integrate current knowledge on the understanding of the correlation between hepatic flow changes and downstream functional changes in the context of liver surgery and interventions. Scale-bridging computational models, which quantitatively describe hepatic anatomical and biochemical data based on image or functional analysis, more and more support understanding. They serve as a tool to relate structure and function and their interdependent changes with the perspective to predict functional outcome after liver surgery (Christ et al., 2017).

The article by Christ et al. summarizes imaging methods and functional assays currently in use for pre-surgical planning. These essentially map selected data sets to whole organ function, which might not be a valid approach due to the multiscale organization of the liver. The authors suggest to link multiscale computational models and experimental and clinical patient data sets in combination with uncertainty assessments in order to reliably predict functional outcome after liver surgery. As one example, Köller, Grzegorzewski, König integrated indocyanine green (ICG) clearance assay data into a physiologically based pharmacokinetic (PBPK) model to predict changes in ICG pharmacokinetics in the cirrhotic liver and after liver resection. The ICG assay is regarded to reflect overall liver function in general. It is highly dependent on individual patient data like age, gender, body weight besides others. Moreover, pathophysiologically relevant patient-specific features like hepatic blood flow and volume, cardiac output, or bile acid transporter abundance may have an impact on liver function. Therefore, all these parameters were included into the PBPK model of ICG clearance to tailor model predictions on patients' need of individual therapy stratification and surgery planning. Indeed, their model allowed for the estimation of individual patient survival. Perspectively, this model shall be integrated into a software application to support pre-surgery planning (Köller, Grzegorzewski, Tautenhahn et al.).

Patient-tailored surgical planning today mainly relies on the determination of the liver volume computed from imaging data, assuming that liver volume represents liver function. This, however, may not meet reality. As mentioned above, individual patient features may impact on liver function not reflected by mere imaging data. Therefore, recent activities increasingly aim to develop imaging technologies that allow for insights into the hepatic structure-function relationships. Two frequently used technologies, magnetic resonance imaging (MRI) and elastography (MRE) are based on the evaluation of biomechanical properties of the liver. They may unravel spatio-temporal resolution of hepatic blood flow, volume and velocity of flow (Bane et al., 2019).

MRI does not only allow for accurate reconstruction of the actual healthy and diseased hepatic anatomical architecture, but may also be used to parameterize continuum biomechanical structure-function models. In particular, spatially inhomogeneously distributed poro-viscoelasticity is an essential mechanical parameter in this context. Still, the measurement protocols as well as model assumptions are not standardized, and a wide range of parameter assessments can be found in the literature. Seyedpour et al. therefore designed a systematic review to reflect the current status quo of MRI-based parameter determinations. Along this line, mDIXON MRI fat quantification data were translated into patient-tailored computational models of the liver to simulate biophysical material (tissue) properties and thermal dose application for patient-specific prediction of microwave ablation therapy (Servin et al.).

Based on the versatile availability even in smaller hospitals, computer tomography (CT) is a common modality to decipher tissue abnormalities prior to liver surgery. Segmentation is used to reconstruct 3D structures based on 2D image sections, which today involves automated or semi-automated procedures (Christ et al., 2017). This, however, may deliver incomplete or even false information because of intra- and extrahepatic low contrast differences. Jiřík et al. propose to include reliably segmentable body key position points like, e.g., the spine and the body surface into a convolutional neural network widely used for image segmentation. The addition of positional information thus improves organ boundary definition and in turn liver segmentation of CT scans.

The liver is not an isolated organ, but communicates with other organs via the vascular system. It is thus prone to turbulences in blood pressure homeostasis. Consequently, inclusion of the vasculature may reflect this inter-organ communication. Modeling the hepatic vascular tree attempts to relate flow changes to function, integrating a higher order of systemic complexity. Torres Rojas et al. outline this by presenting a model of the hepatic vasculature featuring a deterministic architecture delineated from the constructal law of design spanning from the macro to the microcirculation scale. Based on a multi-organ ordinary differential equations model of zonated hepatic energy substrate metabolism (Ashworth et al., 2016), Verma et al. propose another higher level of complexity. Their model includes regulation of glucose metabolism by intrahepatic nerves and the extrahepatic central nervous system in addition to cell-cell communication via calcium, a major stimulant of glucose production from hepatic glycogen.

In summary, mathematical modeling may capture liver function on different scales ranging from intrahepatic molecular clues to superordinate inter-organ communication resulting in a systems view on liver function regulation. The papers contributing to the Research Topic presented here give an overview on approaches to assess liver function by multidimensional model simulations. Inclusion of predictive tools based on such models together with individual patient data into the clinical pre-operative planning routine may overcome the shortcomings of current options and may represent a step forward to a personalized patient-tailored liver surgery planning.

## Author Contributions

All authors listed have made a substantial, direct, and intellectual contribution to the work and approved it for publication.

## Conflict of Interest

The authors declare that the research was conducted in the absence of any commercial or financial relationships that could be construed as a potential conflict of interest.

## Publisher's Note

All claims expressed in this article are solely those of the authors and do not necessarily represent those of their affiliated organizations, or those of the publisher, the editors and the reviewers. Any product that may be evaluated in this article, or claim that may be made by its manufacturer, is not guaranteed or endorsed by the publisher.
